# An Intelligent Healthcare System Using IoT in Wireless Sensor Network

**DOI:** 10.3390/s23115055

**Published:** 2023-05-25

**Authors:** Tallat Jabeen, Ishrat Jabeen, Humaira Ashraf, N. Z. Jhanjhi, Abdulsalam Yassine, M. Shamim Hossain

**Affiliations:** 1 School of Interdisciplinary Engineering & Sciences (SINES) NUST, Islamabad 44000, Pakistan; tallat_cs@yahoo.com (T.J.); ishrat.jabeen@sines.nust.edu.pk (I.J.); 2 Department of Computer Science and Software Engineering, International Islamic University, Islamabad 44000, Pakistan; humaira.ashraf@iiu.edu.pk; 3 School of Computer Science, SCS Taylor’s University, Subang Jaya 47500, Malaysia; 4 Department of Software Engineering, Faculty of Engineering, Lakehead University, 955 Oliver Rd, Thunder Bay, ON P7B 5E1, Canada; ayassine@lakeheadu.ca; 5 Department of Software Engineering, College of Computer and Information Sciences, King Saud University, Riyadh 11543, Saudi Arabia

**Keywords:** genetic algorithm, cryptography, wireless sensor networks, authentication, IoT, remote patients monitoring system, intelligent healthcare

## Abstract

The Internet of Things (IoT) uses wireless networks without infrastructure to install a huge number of wireless sensors that track system, physical, and environmental factors. There are a variety of WSN uses, and some well-known application factors include energy consumption and lifespan duration for routing purposes. The sensors have detecting, processing, and communication capabilities. In this paper, an intelligent healthcare system is proposed which consists of nano sensors that collect real-time health status and transfer it to the doctor’s server. Time consumption and various attacks are major concerns, and some existing techniques contain stumbling blocks. Therefore, in this research, a genetic-based encryption method is advocated to protect data transmitted over a wireless channel using sensors to avoid an uncomfortable data transmission environment. An authentication procedure is also proposed for legitimate users to access the data channel. Results show that the proposed algorithm is lightweight and energy efficient, and time consumption is 90% lower with a higher security ratio.

## 1. Introduction

The twenty-first century has seen quick changes in social standards, commercial practices, and technology. There has been an industry revolution known as the fourth industrial revolution because of most industries shifting towards automation and a reduction in the amount of human input (Industry 4.0). Industry 4.0 (IR 4.0), also referred to as the fourth industrial revolution, depends on IoT and wireless sensor networks (WSN). Several control systems, including those that monitor the environment, automatically match homes, and find chemical and biological threats, use IoT and WSN. IoT devices and apps process extracted data from WSN devices before being sent to distant places. This evaluation identifies research gaps, provides a wealth of information on Industry 4.0, and suggests future initiatives [1].

WSNs (Wireless Sensor Networks) are used in a range of applications. The most well-known applications include target tracking, habitat monitoring, surveillance, and security [2,3]. Sensor nodes in a WSN are small, battery-powered, and inexpensive. One of the key issues for a WSN is energy usage during communication between sensor nodes. Through a radio transmitter called sink, the detector or sensor device communicates the moderately managed information to a control section. A base station is the name given to the sink (BS). The sensor points deliver information to the sink directly or through gateways. This gateway acts as a processed information collection point. Nano sensors are usually inimitable once placed, and they also have application-particular needs. Among the various types of sensors are optical, multimedia, thermal, undersea, acoustic, magnetic or electromagnetic [4], biological, chemical, seismic, mechanical, or vehicle movement sensors [5].

A wireless sensor network is made up of many wirelessly connected sensor nodes. The sensor nodes work together and can self-organize. WSN architectures are able to be either structure-less or deterministic. WSN applications include a wide range of monitoring and tracing applications. These include habitat monitoring, environmental monitoring, surveillance, military, public, industrious, health monitoring, business, and multimedia monitoring. WSN tracking applications are distributed as two categories of military and habitat. One of the main concerns of experts working in the field of wireless sensor networks (WSNs) is secure information routing, since sensor nodes collect data from the physical environment and transfer them to base stations for authorized users to access over the internet. Most often, sensor nodes in a WSN are placed in unsupervised areas with the potential for allowed or unauthorized access. The necessity to protect transmitted information from hackers has arisen because of these and other factors [6].

The most difficult aspect of creating wireless sensor networks is ensuring network coverage. By examining network coverage to determine if there is a communication blind region and obtaining the monitoring area of wireless sensor network coverage [7,8,9,10], the network coverage must reach its maximum under conditions that deliver a certain level of service (QoS). Depending on the measurement condition, the sensor node distribution may be changed, or new sensor nodes can be added. Extra sensor nodes have also been installed in crucial locations by changing the network coverage density to ensure that the data gathered were correct. As a result, network coverage is critical not only for communication and monitoring, but also for meeting the requirements of certain applications.

**Figure 1:**
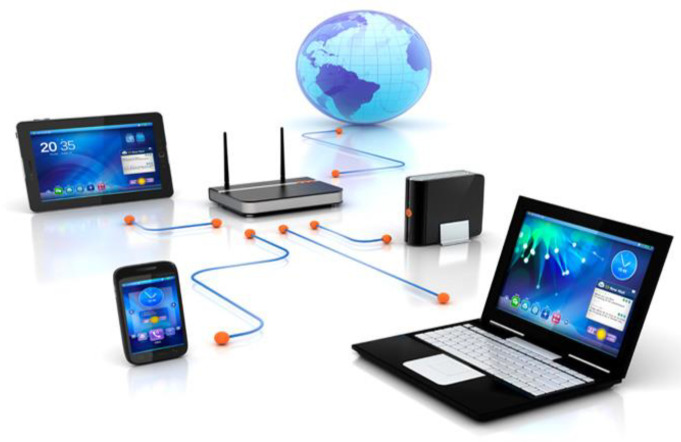
Architecture of wireless sensor networks.

Figure 1 shows the architecture of a wireless sensor network where the data is transferred from a personal device to the access point. Internet cloud relates to the access point where the internet connection is developed, and the data are transmitted towards the server.

Data security is the main concern in wireless data transmission, as lots of hackers and data manipulators can easily affect smooth transmission. Therefore, multiple data security algorithms are developed to ensure data protection. These algorithms, however, have certain drawbacks. When the transmission data are considerable, the Huang algorithm’s complexity is too great; it is also a common cause of channel obstruction. Although the Tian D algorithm has a lower computing and communication complexity, it has two flaws: first, it is unable to render redundant nodes at the monitoring area boundary inert, and it also fails to address node coverage area overlap, resulting in the election of too many working nodes, limiting the network’s lifetime. In high-density sensor networks, the CCP technique fails [11].

Based on the issues and the unique predominance of WSNs, a WSN node optimal coverage method based on improved genetic algorithm is proposed. This method uses the location information of the nodes to build a node coverage optimization model, which is then solved using the improved genetic algorithm.

### 1.1. Contributions and Novelty

- This work introduces a novel cryptography technique based on a genetic-based technique with a logical–mathematical model.
- In this article, security analysis and experimental findings of the proposed technique are presented to validate its efficacy over existing techniques.
- A strong authentication process is proposed to smooth data transmission and avoid malicious node attacks. IoT devices share the sensor data they collect by connecting to an IoT gateway.
- Resultant comparisons of the proposed technique are presented to analyze the time efficiency parameters.
- To explain the most recent developments in intelligent healthcare systems using the internet of things.
- Encourage the transmission of encrypted, safe data via a trustworthy, lightweight route to improve the networks’ performance.
- To evade third party attackers and hackers from accessing the data over the wireless channel.

Encourage efficient encryption of data in terms of cost, processing time, and sensor memory space.

### 1.2. Literature Review

WSNs are self-contained and widely dispersed throughout the universe. Because there is no central authority and nodes are randomly placed in the network, a WSN is subject to security vulnerabilities. A well-known WSN attack is malicious stabbing, in which a hacked node deceives other nodes by imitating one of the network nodes.

Low-power sensor nodes make up wireless sensor networks (WSNs). Without the need for a physical media, these nodes can monitor physical conditions and share information. WSNs are self-contained and widely dispersed in their surroundings. A WSN is vulnerable to security flaws since there is no central authority and nodes are randomly placed in the network. A malicious WSN attack is a well-known WSN assault (such as a compromised node imitating one of the network nodes, thus misleading other nodes). Several techniques have been established in the art of work to tackle these dangers, such as cryptographic processes or time synchronization. These solutions, however, may fail due to WSN’s independent structure. The Hamming residue method (HRM) was proposed by Alotaibi et al. as an efficient way to mitigate malicious attacks. The experimental results back up the proposed method [12].

Since sensor nodes collect data from the physical environment and transmit them to base stations for authorized users to access through the internet, experts around wireless sensor networks (WSNs) are worried about safe information routing. Sensor nodes in a wireless sensor’s networks are often placed in unattended areas where permitted or unauthorized access is possible. This has raised the need to secure transmitted data from outsiders, among other things. Cryptography has been chosen as the most effective approach for achieving the three major security needs of data secrecy, authentication, and integrity, even though there are various techniques for securing a network (CIA). In this work, the article [13] presents an overview of current WSN challenges as well as potential solutions to these issues. To solve the security problem posed by present WSN systems, Salau et al. offered efficient and resilient encryption and decryption algorithms for secure communication. The most notable aspect of this system is that it uses both substitution and transposition cypher methods to create encrypted text. The approach was created after thoroughly examining existing algorithms in this field [14].

Due to its portability, low cost, and ease of implementation, a Wireless Sensor Network (WSN) is one of the best techniques for many real-time applications. Monitoring the area of interest, gathering data, and sending them to the base station (Access point) for post-processing and analysis are the duties of the WSN. Some WSN implementations make use of a lot of sensor nodes. Additionally, the battery life and memory of these wireless nodes are constrained. Therefore, to maximize the benefits of these WSNs, these WSN nodes must have a management system capable of controlling their interactions with one another and the access point [15].

Introducing new technology without considering security problems has shown itself to be quite risky. Wireless sensor networks (WSNs), particularly those utilized in sensitive data monitoring applications, are also affected (e.g., health care applications). Despite tremendous progress in resolving several weaknesses in sensor networks, such as coverage difficulties, power constraints, and making the most of limited network capacity, sensor network security is still in its early stages. Due to a variety of resource restrictions on individual sensors, WSN security confronts numerous well-known challenges. The need to safeguard these networks is growing more pressing. Symmetric key cryptography is frequently thought to be impossible; however, public key cryptography has its own issue with key distribution. Based on these authors’ earlier work, which includes UES versions II and III, this study proposes a new symmetric encryption standard method. UES version-I, Modifield UES-I, UES version-II, and UES version-III are some of the most efficient encryption algorithms recently developed by Roy et al. The Ultra Encryption Standard version-IV algorithm is the name of the algorithm. It is a bit-wise reshuffle and bit-wise columnar transposition multi-encryption symmetric key cryptosystem [16].

Wireless sensor networks (WSNs) are made up of self-contained sensor nodes that are connected to one or more base stations. One of the primary purposes of wireless sensor networks is to transport trustworthy data from one node to another. As wireless sensor networks expand, they become more vulnerable to attacks, necessitating the implementation of robust security systems. Due to the sensor nodes’ limited energy, processing, and storage resources, finding appropriate cryptography for wireless sensor networks is a significant task. We used the AES (Advanced Encryption Standard) encryption technique in this paper to provide acceptable degrees of security for ensuring the confidentiality of data in the WSN network [17].

Cryptography has been chosen as the most effective approach for achieving the three major security needs of data secrecy, authentication, and integrity, even though there are a range of techniques for securing a network (CIA). We offer an overview of current concerns in a WSN, as well as potential solutions to these issues, in this study. To solve the security problem posed by present WSN systems, we offer efficient and resilient encryption and decryption methods for secure communication. The most notable aspect of this system is that it uses both substitution and transposition cypher methods to create encrypted text. The approach was created following a thorough examination of current methods in this field [18].

Multiple other techniques are presented, including fault tolerance detection [19] and extending directed diffusion routing algorithm [20] in wireless sensor networks.

Sensing networks are used in many fields; therefore, the security of transportation of data is the bigger concern [21]. The authors used many techniques to secure the data, being transmitting, end-to-end data transfer [22], and hop-to-hop TCP [23] towards server is also projected.

Various networking schemes e.g., Ad hoc networking [24] and underwater networking [25] are used in many fields to transfer data from one place to another.

Zhang et.al explain that the widespread use of wireless sensor networks creates a strong foundation for the growth of the internet of things. It is vital to research wireless sensor network security to lessen security threats and network attacks, since the node deployment environment of wireless sensor networks is typically quite complex. The security and use of the wireless sensor network are highlighted [26].

Another article [27] used a technique for synchronization of data security and [28] used quantum key distribution to secure the data from malicious nodes.

IoT (Internet of Things) entities gather substantial sensory data and send them to the cloud for analysis. IoT faces a huge problem in integrating resource-constrained, lossy, and low-power devices that serve a variety of sensitive applications with requirements into reliable and secure data aggregation and forwarding [28].

It takes more time and effort to find security concerns, consider the volatility of IoT environments, and offer automated solutions. Network security is crucial on the Internet of Things (IoT), especially since packets must travel through heterogeneous networks made up of billions of intelligent nodes. It takes a lot of study to secure the routing process between various IoT devices across heterogeneous networks. Network layer security is one of the major information security issues in IoT [29].

The rest of the sections are structured as follows:

Section 1 presents the proposed studies’ introduction and related literature, Section 2 presents the proposed materials and methodology, and Section 3 presents the security analysis of various attacks. Results and discussion are explained in Section 4. Section 5 concludes the studies.

## 2. Materials and Methods

The suggested approach is given in this part, which includes an encryption algorithm and an authentication mechanism. The data transmission protocol is also detailed, along with mathematical modelling, to demonstrate the system in full.

Wireless sensors detect data, after implementation of encryption algorithm data, are transmitted towards a server through a wireless channel.

**Figure 2:**
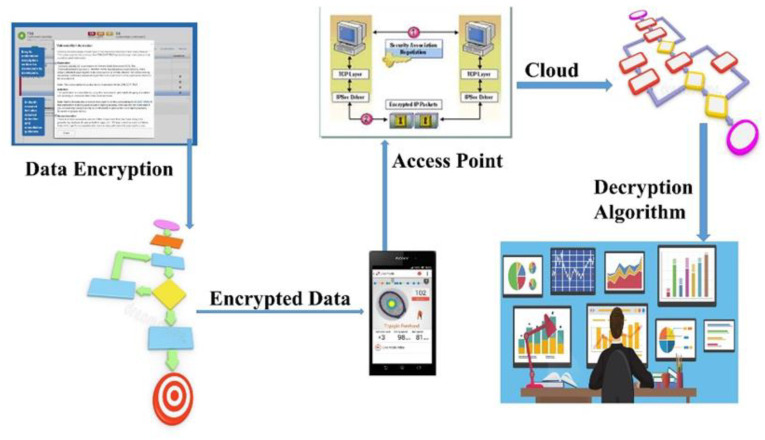
Conceptual Workflow of Proposed Genetic-based System on WSN.

To secure the patient’s data through WBAN, a lightweight genetic-based encryption technique is proposed. The suggested encryption technique is used to turn the data into ciphertext after identifying the patient’s health data. Figure 2 shows how encrypted data are transferred to the collector, who then sends it to the medical server across the network.

**Figure 3:**
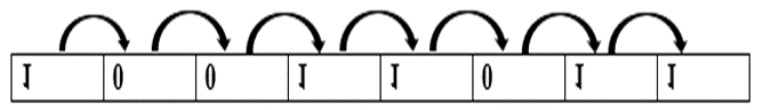
Example String 1.

The proposed algorithms take 8-bits of plaintext data to perform security algorithms steps. Plaintext is first transferred into the decimal conversion and then in binary converter is applied over the data because plaintext data should follow all the conversion steps accordingly over the sensors. After binary conversion, 8 bits of binary perform XOR operation with the key generated by the key generation algorithm. Crossover is executed over the temporary production of the new bits. In the crossover, the right side of the 4 bits are swapped at the left side, and the left side of the 4 bits are swapped towards the right side, vice versa. The produced 8-bits are the genetic bits from which the chromosome bits are generated by performing the circular operation, as shown in Figure 3. Starting from the crossover to the chromosome’s bits, a loop is executed until two times, and after completing its circle reproduction, bits are extracted from the above loop execution.

Reproduction bits are the alphabetical order bits that are performed over the all-possible pairs of 4 bits each, as shown in Table 1. Two pairs make 8-bits of data, and the substituted two alphabets are the ciphertext of the data, which is very unpredictable for a third party over the wireless network. Chromosome bits are the strings of data that will be converted into the reproduction bits of alphabetical form by a substitution process.

**Figure 4:**
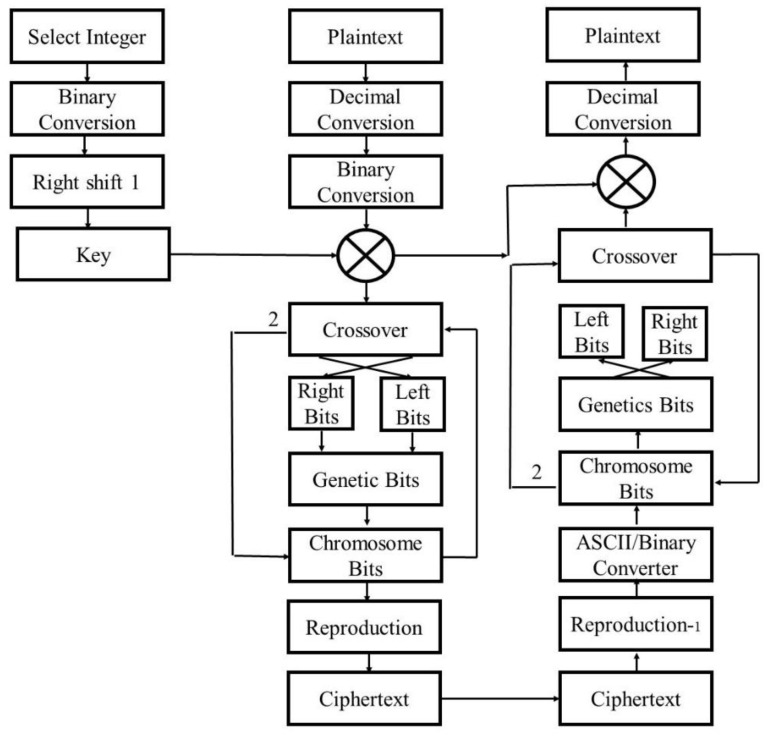
Genetically Cryptographic algorithm for data security.

The whole process of the cryptographic algorithm is shown in Figure 4. The decryption process follows all the designed steps backwards at the receiver ends.

The algorithm is simple and efficient because there are fewer steps in this encryption technique. The key creation algorithm similarly uses simple mathematical processes to generate an 8-bit private key, which is then used in the genetic encryption technique. Figure 4 presents the private key algorithm which is shared with the server and user only. The proposed algorithm uses private–private key algorithm for the transmission of encrypted data.

Authentication is the method through which a person or computer verifies their identity with a server or client. A username and password are frequently required to authenticate a server. Cards, retina scans, voice recognition, and fingerprints are examples of other means of authentication.

**Figure 5:**
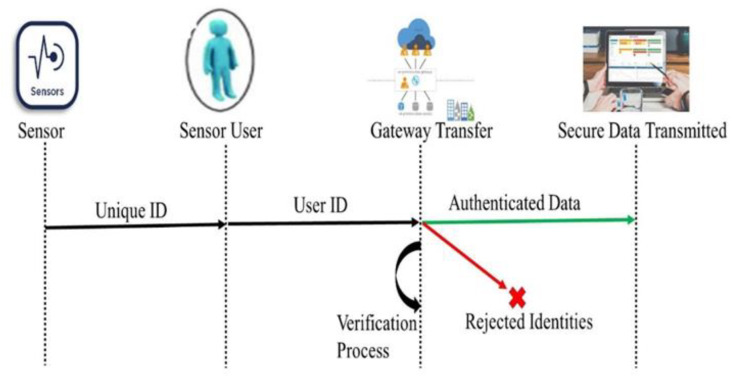
Authentication Process for Smooth Data Transmission.

In Figure 5, sensors that are used for data detection must be registered with the user, and the user’s ID with the sensor’s ID is sent towards the gateway to match the authentication ID. Then, after verifying the data sources, data are sent towards their destination and the data from the fake identities are rejected.

### Mathematical Modelling

Mathematical modelling for the genetic-based algorithm is explained. P is the plaintext which is converted into decimal numbers D, and then binary conversion B is applied.

(1) $$P=\sum_{D}B$$

B′ are the temporary resultant bits after performing the XOR operation between the key generated k by the lightweight process and the binary converted bits B. This is example 2 of an equation:

B′ = K⨁B(2)

8-bits binary are divided into two halves, first four bits are denoted as B′L left side binaries and the next four right side binaries are denoted as B′R.

(3) $$C=\frac{B^{\prime }}{2}$$

C′ = B′L + B′R(4)

C′ resultant bits are generated from the above operations and a crossover circulation process is applied for the genetic bits Gb. Cb are chromosomes bits.

C′ = Gb σ Cb(5)

The chromosome’s bits Cb are reproduced Rep from the reproduction Table 1, where bits are converted into alphabetical strings, and this is the final step to generate ciphertext Ci.

Cb = XRep(6)

XRep = Ci(7)

## 3. Security Analysis

Public key cryptography can prevent insider attacks, but it is too expensive for network use. The routing protocols in a WSN network has a distinct identity. In this scenario, various attacks are avoided by the proposed system to maintain data security.

### 3.1. Blackhole

A black hole attack occurs when a malicious node captures all data and responds falsely to any route request, preventing the source node from communicating with the destination node, since it lacks an active route. Equation 8 shows that node A and node B are legitimate users and want to communicate with each other; however, the malicious node captures the transferred data between node A and node B.

X(A,B) = Z(XA) − Z(XB)(8)

The approach we present is based on a new routing algorithm that analyses the shortest path to prevent harmful node paths. Our findings show that our proposed routing technique is successful and efficient, as only legitimate users are allowed in the system after the authentication process.

### 3.2. Selective Forwarding

The selective forwarding attack is a type of packet drop attack that impairs the performance of a wireless sensor network by dropping sensitive packets received from sensors. In an open wireless environment, it is difficult to detect. Equation (9) presents that data transmission between node A and node B is interrupted, and the data spoofing technique is used by the attacker node Z.

X(A,B) = Z (X(A) + X(B))(9)

The recommended approach deals with this type of attack by using an authenticated user ID with the packet transmitted by the legitimate user, and the packet without authenticated ID is dropped by the receiver.

### 3.3. Sybil Attack

A Sybil Attack is a massively disruptive attack on a sensor network in which many legitimate identities are used in conjunction with falsified identities to gain unauthorized access to a network. A Sybil attack is when a node pretends to be someone else to other nodes. In Equation (10) an attacker node Z pretends to be an authenticated node and disturbs the smooth data transmission.

X(A,B) = Z(A,B)(10)

The outsider attack can be blocked using authentication and encryption techniques by mounting a Sybil assault on the sensor network. The proposed techniques stopped the Sybil attack by employing identity validation, and the authentication procedure will prevent this assault.

### 3.4. Hello Flood Attack

The most prevalent network layer attack is the hello flood. Many nodes, even those far away in the network, choose a node broadcasting a high-powered Hello packet as the parent node, resulting in Hello flood assaults. Equation 11 demonstrates the hello flooding attack as the attacker node Z flows hello flooding between the sender node A and receiver node B to disrupts the transmission.

X(A,B) = Z(X(A)),Z(X(B))(11)

To prevent the hello flood attack, a cryptographic approach with authentication process is utilized. The secret key is shared by all sensors. During the transmission, a new and simple efficient encryption key is generated to avoid the third person, as the authenticated nodes share the secret key.

## 4. Results and Discussion

Sensors detected the real time health data of patients and converted them into cipher text which was transferred into the wireless channel to a doctor or hospital server. The proposed genetic-based algorithm with an authentication process was tested over MATLAB. The results were tested over time complexity parameters and attack avoidance.

The amount of time it takes for a certain method to perform a computing operation is known as computation time or running time. The time complexity parameters that dis-played its efficiency in this research are the time consumed by the encryption method coupled with key generation processes.

### 4.1. Encryption Time Complexity

**Figure 6:**
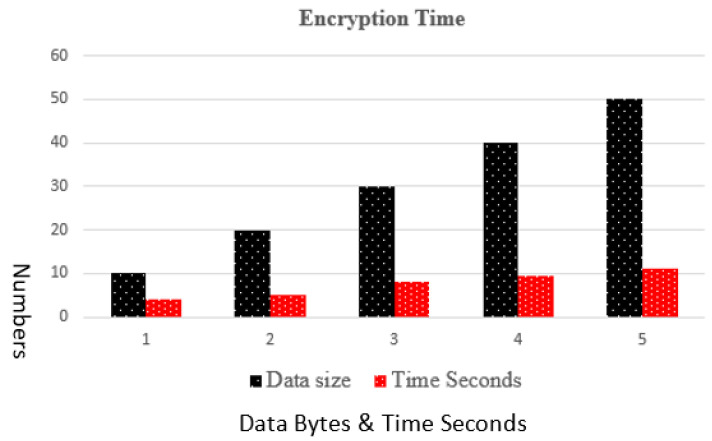
Encryption Time Complexity.

Encryption time complexity is the running time the proposed algorithm takes to execute data bytes. Figure 6 shows that the proposed algorithm executes data bytes in less time slots, and as the number of data bytes increased the time taken by the proposed algorithm is also enhanced. Overall, the time complexity remains lesser.

### 4.2. Decryption Time Complexity

**Figure 7:**
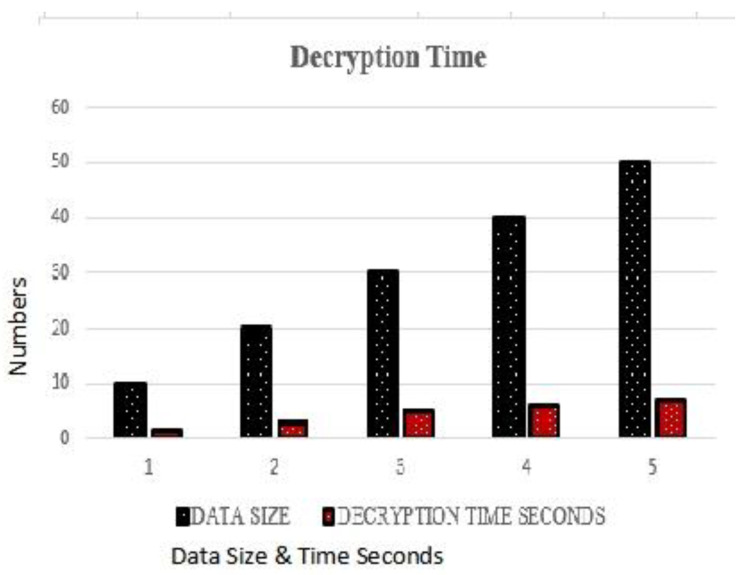
Decryption Time Complexity.

Decryption time complexity is the time taken by the algorithm to decrypt or to unwrap data at the server side. Figure 7 presents the decryption time values with respect to the data bytes. Decryption time is enhanced as the data bytes for execution are increased, but overall, it is observed that the proposed algorithm took much less time to decrypt the data.

### 4.3. Comparison Analysis of Encryption Time Complexity

**Figure 8:**
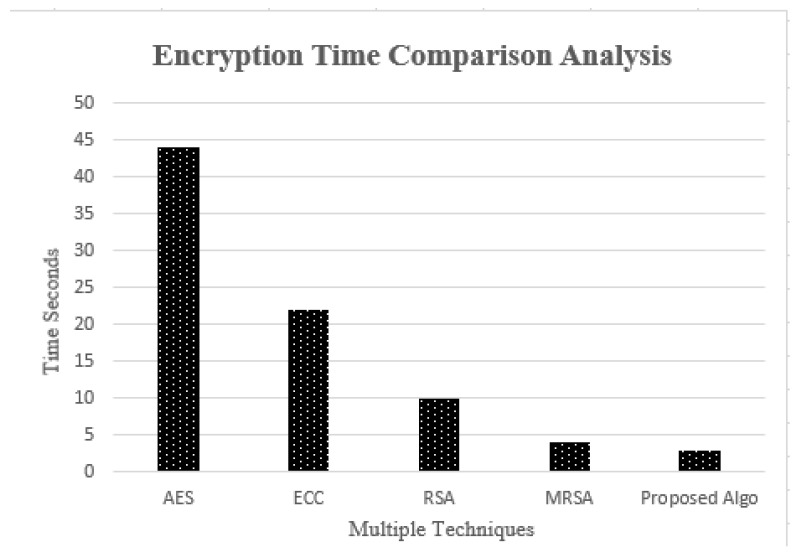
Comparison Analysis of Encryption Time Complexity.

Comparison Analysis of the encryption time complexity was determined with respect to the various existing encryption techniques. Figure 8 presents the time complexity comparison for encryption analysis with respect to various existing schemes [27,28]. It is reflected that the proposed genetic-based encryption algorithm time complexity is less than when compared to the existing techniques.

### 4.4. Comparison Analysis of Decryption Time Complexity

**Figure 9:**
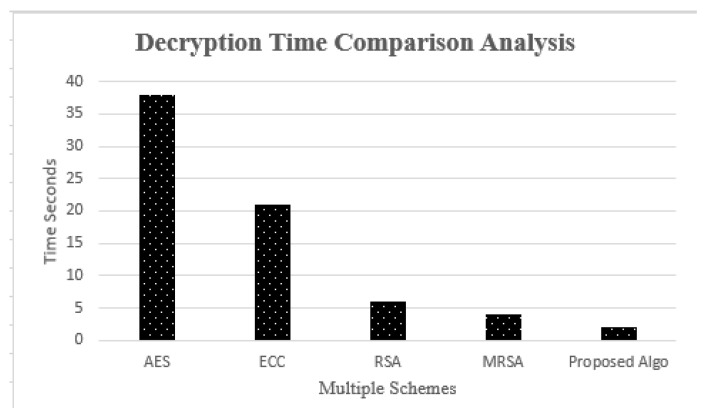
Comparison Analysis of Decryption Time Complexity.

In [27,29], the suggested decryption algorithm’s time complexity was compared to that of existing approaches. Figure 9 illustrates that when compared to the other methods, the proposed algorithm takes less time to unwrap data by the receiver.

Wireless data transmission needs lightweight security procedures to transfer data successfully from one place to another. Lightweight security algorithms could be helpful to avoid various attacks on data transmission over the wireless channel; however, time complexities for the security algorithm should also be lesser [27,28]. It is demonstrated that if the algorithm complexity is lesser, it is easy to use, and its implementation cost is also less. Cost is minimal as there is no need for special hardware, and support is easily handled remotely. In practical life, people can easily adapt to an intelligent healthcare system for regular checkups to avoid hospital visits.

### 4.5. Comparison Analysis of Total Cost Computation

**Figure 10:**
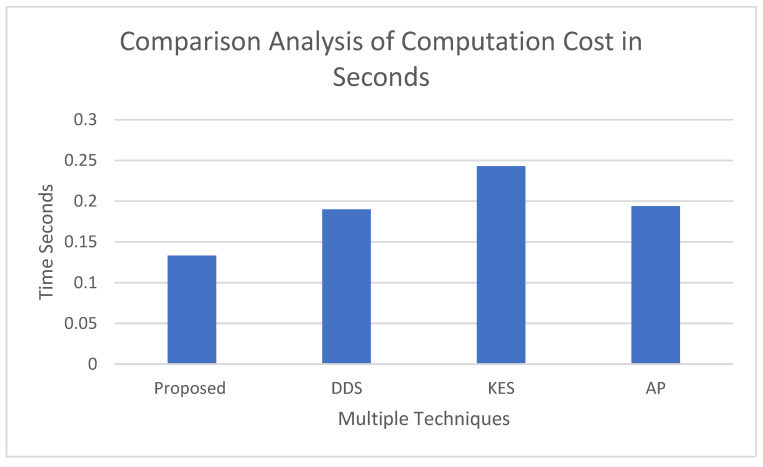
Comparison Analysis of Total Computation Cost of System.

Figure 10 presents that the total computation cost of the proposed technique is less than when compared to the literature techniques in digital design signature [27], key establishment scheme KES [28],and authentication protocol AP [29]. Total computation cost (TCC) is compared in seconds, and the proposed system can be implemented easily.

## 5. Conclusions

Wireless sensor networks are networks of spatially dispersed and specialized sensors that monitor and record environmental factors before transmitting the information to a central location. There are still numerous obstacles to overcome in terms of data security and privacy. Although the cloud provides access to data storage with flexibility and ease of management, there are still opportunities for unauthorized attacks and criminal actions. Sensitive data could be stored discreetly on the cloud server. As a result, data security is a top priority. The proposed method of a genetic-based algorithm increases the lifetime of the system. The proposed system uses a secure cryptographic technique with an authentication procedure to ensure smooth data transmission and avoid malicious node attacks. Results were analyzed through the prevention of various attacks on the data. According to the simulation results, utilizing a GA-based technique extends the network’s performance as compared to the existing techniques.

Future technologies can merge the healthcare system with some artificial intelligence models to make the system more effective and intelligent.

## Figures and Tables

**Table 1:** Reproduction.

Chromosomes Bits	Reproduction	Chromosomes Bits	Reproduction
0000	T	1100	P
0001	E	1101	S
0010	Y	1110	U
0100	A	1010	V
0101	H	1011	W
0110	R	1001	X
0111	M	1111	Y
1000	D	0011	Z

## Data Availability

Data will be available on request.
